# Active Tracking of *Maja Squinado* in the Mediterranean Sea with Wireless Acoustic Sensors: Method, Results and Prospectives

**DOI:** 10.3390/s131115682

**Published:** 2013-11-15

**Authors:** Jean-Sebastien Gualtieri, Antoine Aiello, Thierry Antoine-Santoni, Bastien Poggi, Emmanuelle DeGentili

**Affiliations:** 1 University of Corsica/UMS CNRS STELLA MARE 3514, Lieu-dit U Casone, Lido de la Marana 20620 Biguglia, France; E-Mail: aiello@univ-corse.fr; 2 Société Informatique et Télématique Corse (SITEC)/Avenue Paul Giacobbi 20600 Bastia, France; 3 University of Corsica/UMR CNRS SPE 6134, Quartier Grossetti 20250 Corte, France; E-Mails: antoine-santoni@univ-corse.fr (T.A.-S.); bpoggi@univ-corse.fr (B.P.); gentili@univ-corse.fr (E.D.)

**Keywords:** active tracking, wireless acoustic sensor, method, *Maja squinado*

## Abstract

The Sustainable **TE**chnologies for **L**ittora**L A**quaculture and **MA**rine **RE**search (STELLA MARE) platform has as an objective to provide data for the management of the sea in relation with the fishing industry. In this paper, we introduce the first experiment on the active tracking of a crab species, *Maja squinado*, symbolic of the deregulation of fishing activity. This paper introduces the method used for monitoring *Maja squinado* and the first collected data on the behavior of this little-known species.

## 1. Introduction

The global changes which affect the Earth have more important consequences in closed spaces. This is the case of the Mediterranean Sea, and especially in the island area. In relation to this subject, in 2010 the University of Corsica decided to create the **S**ustainable **TE**chnologies for **L**ittora**L A**quaculture and **MA**rine **RE**search (STELLA MARE) scientific platform. This research center has been certified by the National Centre for Scientific Research (CNRS), and this platform became the Mixed Services Unit (UMS) 3514 Stella Mare University of Corsica/CNRS. This unit is specialized in Marine and Littoral Ecological Engineering. In this way, we have defined, in interaction with the professional fishing industry, species to be studied both in laboratories and *in situ*. In this paper, we present our works focused on the behavioral of crustacean species, more particularly the case of *Maja squinado*. This species, very appreciated (real economic interest), is now weakened and protected by order n° 323/2004/DRAM. Better knowledge of the behavior of this crustacean family could help define a strategic protection and development scheme. To obtain our results, we have used an active tracking tool. This monitoring allows us to cross a first step in the follow-up of *Maja squinado* in real situations. Furthermore, this methodology could be applied to others species like lobsters (*Homarus gammarus*), spiny lobsters (*Palinurus elephas*), and also fishes.

Our objective is the modeling and simulation of the behavior of the *Maja squinado*. However we don't have enough data to build a model. The first step of this project is to monitor the behavior of this species based on acoustic telemetry and build a database. Our study of this species is based on the work already done in Galicia (Atlantic coast of Spain) by González-Gurriarán [1–3].

Numerous behavioral studies of marine species have led to a rapid development of new applications for acoustic telemetry [4–6]. To learn more about the life cycle of an aquatic species, resource utilization and its ecological role in an ecosystem, it is essential to accurately track its movements more or less long term [6,7].

Over the past 30 years, acoustic telemetry providers have developed different approaches to medium baseline high resolution acoustic positioning systems. Active tracking and passive acoustic monitoring have become powerful tools to quantify complex movements on different spatial and temporal scales (e.g., [6,8–10]). While active monitoring can provide precise movement information on a few individuals in a short period, passive monitoring is used to quantify the information of several individuals (presence-absence data) in the longer term. Although less expensive in terms of resources and time, passive monitoring don't allow data acquisition and position tracking as accurately as active tracking [10,11]. However, the new positioning systems allow one to accurately track several individuals [12–14], but a monitoring program using hydrophones in a delimited area, implies that we already know the behavior of the studied species. In our case, we can't use this system because we don't know if spider crab would stay in the same area or travel great distances.

The paper is organized as follows: in Section 2, we introduce some information on the studied species, materials and methods of these series of experiments. Section 3 shows the results under different use configurations. In Section 4, an analysis and a critique of the results are presented, and we conclude the paper in Section 5.

## 2. Experimental Section

### 2.1. Maja Squinado: Species Information

The spider crab *Maja squinado* is, along with the edible crab (*Cancer pagurus*) the biggest crab of European coasts. This explains why it has been mentioned early and many authors interested in marine life have spoken about it. The first authors to have dealt about the spider crab in some way were Rondelet (1554) and Aldrovandus (1606). Herbst was one of the first to describe in detail the spider crab (1788).

The general idea that emerges from the literature is that this species has an omnivorous diet. Animals adapt to the environment in which they find themselves [15,16]. This type of behavior seems appropriate for adult animals that have to migrate over long distances across different biotopes. However, it is possible that the juvenile stage of life, the environment is more stable and the diet is narrower.

The literature shows that we can find *Maja squinado* on all types of sea bottoms. Several authors have noted that, due to migration, the substrate is different depending on the time of year. However, the information on the links between substrate and season are not consistent between authors:

- Coralligenous in winter, muddy in summer [17,18]
- Rocky in winter, seagrass beds and sandy bottoms in the spring [19]
- Sandy or sandy-muddy throughout the year, except rocky in late spring [20,21]
- Rocky or gravel in winter, grass and sandy or rocky in the spring [22].

Regarding the depth habitat of *Maja squinado*, most authors give a range of 0 to 10 m (variable, in fact, depending on the bathymetry of the area concerned). The deepest catches cited in the literature are 120 m in South Brittany [23], 145 m in Israel [24], 170 m in the Adriatic [25], and 600 m in the Ligurian Sea [26]. Many authors have noted, in the Mediterranean and Atlantic, a deep change according to the time of year due to migration: deep areas in winter, coastal areas in spring and summer. This research took place in the Mediterranean Sea, on the Corsican coast. The study was carried out within the San Fiurenzu fishing cantonment (on the west coast of Cap Corsica: 42°45′N 9°18′E), between March 11th, 2013 and May 3th, 2013.

### 2.2. Used Materials: VEMCO

Manual tracking was conducted from a boat equipped with a VH165 omnidirectional hydrophone (VEMCO Ltd., Bedford, NS, Canada) and a VR100 receiver (VEMCO Ltd.) to locate and track ultrasonic transmitters.

At sea, the accuracy of the manual-tracking method depends on several factors. Firstly, the depth of the transmitter and the presence/absence of a thermocline act as an acoustic barrier. Secondly, vegetation, rocks, waves (in severe weather conditions), and others obstacles and interferences can reduce the power of the signal. At the same study site, it was estimated that the accuracy of the fixes ranged from 10 m to 50 m.

Each position, manually taken, was recorded with date, time, crab ID and signal power. Those coordinates were transformed to geodetic coordinates (WGS84) and interpreted as a theme in the QGIS environment (www.qgis.org).

Tags used in this study are V13TP-1L coded transmitters. These transmitters send acoustic pings at 69 kHz that are infrequent and random about an average delay (60 s). This ping train includes an ID number which allows identification of the specific tag along with the sensor telemetry data and it is equipped with temperature and depth sensors.

The physical specifications for this transmitter are: 13 mm diameter, 45 mm in length, 12 g weight in air, 6 g weight in water and a power output of 150 dB re 1μ Pa @ 1 m. The acoustic positioning transmitters did not exceed 1.1% of the body weight of the crab (Table 1).

### 2.3. Our Method for Active Tracking

The goal of our work is the study of spider crab behavior. This study involves a phase of *in situ* monitoring. Before equipping a spider crab with a tag, we tested several acoustic transmitters to determine which is best suited to our experiment. We performed seven tests and recorded 308 data points. In this part we present the results of these tests.

#### 2.3.1. First Stage: Test of V9 VEMCO Sensor

We started by testing the transmitter V9-1L (VEMCO Ltd). This small tag seemed to be the ideal solution for the spider crabs monitoring. The V9 specifications are: 9 mm diameter, 24 mm length, weight in air 3.6 g, weight in water 2.2 g and power output 146 dB re 1 μPa @ 1 m. Three transmitters were tested for a period of nine days (August 21th to August 29th, 2012). During the test period, the sea was calm and the water temperature averaged 25 °C.

#### 2.3.2. Second Stage: Test of V13 VEMCO Sensor

After tests on the V9 sensors, we tested the V13TP. These larger size sensors have a stronger signal power. We also opted for transmitters that give the temperature and depth. These data proved essential during tracking. The tests were performed under the same conditions as above.

#### 2.3.3. Third Stage: Monitoring Maja Squinado According to Stages 1 and 2

The goal of our work is to monitor spider crabs in their environment and to collect information about their behavior. Movements provide some information on the species (speed, diel activity, reproduction area, *etc.*). Table 1 below describes the characteristics of the individuals studied.

Reference measurements used in this study are the length of cephalothorax (carapace), from the notch between the two peaks or “horns” of the rostrum to the most posterior point of the shell, in accordance with Regulation EEC No 3094/86 of the Council of European Communities of 7 October 1986 (Official Journal of the European Communities No. L288 of 10/11/86).

## 3. Results and Discussion

### 3.1. Tracking Results

**Figure 1.**
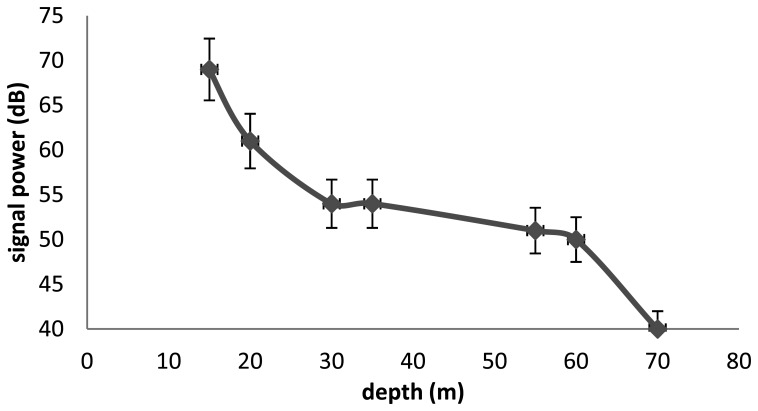
Relation between depth and signal power for V9 tags.

**Figure 2.**
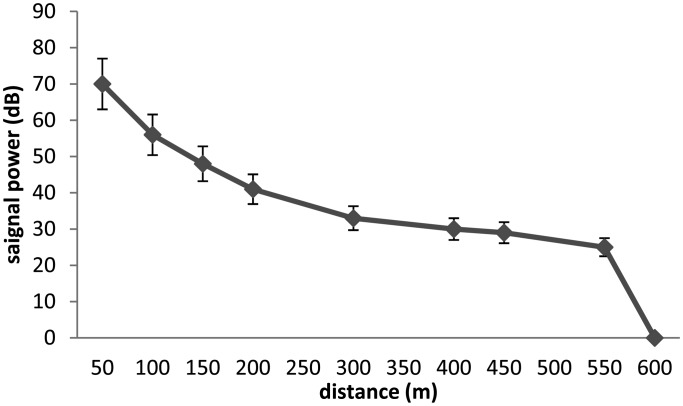
Relation between distance and signal power for V9 tags.

In this part we introduce the different results according to the several stages of tests. Figures 1 and 2 show the capacity of V9 transmitters according to depth and distance to the receptor. The signal is received at the surface using the VH165 omnidirectional hydrophone.

**Figure 3.**
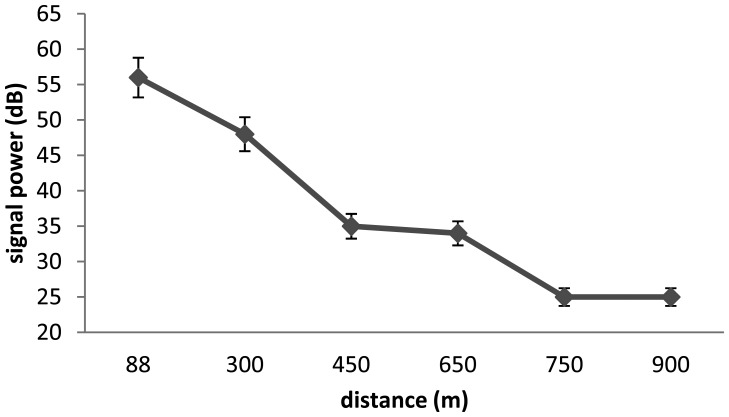
Relation between distance and signal power for V13 tags.

**Figure 4.**
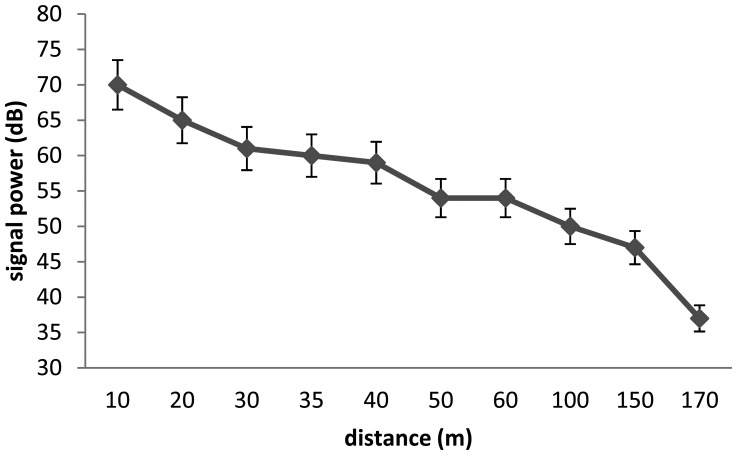
Relation between distance and signal power for a V13 tag under a rock.

In the two cases, we see that the V9 transmitters are limited by the depth and the distance. In Figures 3 and 4 we test the capacity of the V13 tags to broadcast a signal according to two scenari. The capacity of V13 to transmit a message according to the distance to the receptor is illustrated by Figure 3 and the capacity of the transmitters V13 under extreme conditions is shown in Figure 4. In order to better anticipate the behavior of a spider crab, we tested the power of a transmitter under a rock. We were not able to capture the signal from a V9 under a submerged rock. On the other hand, Figure 4 shows that we can capture the signal from a V13 under the same conditions up to 170 m.

**Figure 5.**
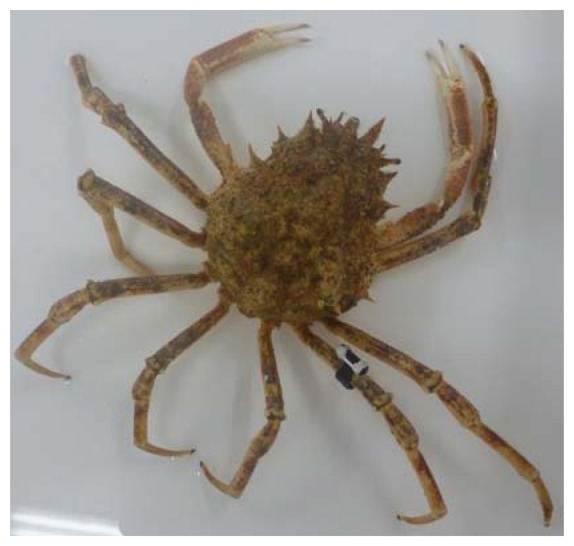
Maja 1 equipped by a V13TP VEMCO transmitter.

According these tests, we decide to equip the spider crab with V13TP VEMCO sensor (Figure 5) and to start an active tracking campaign on an individual Maja 1 during two months.

**Figure 6.**
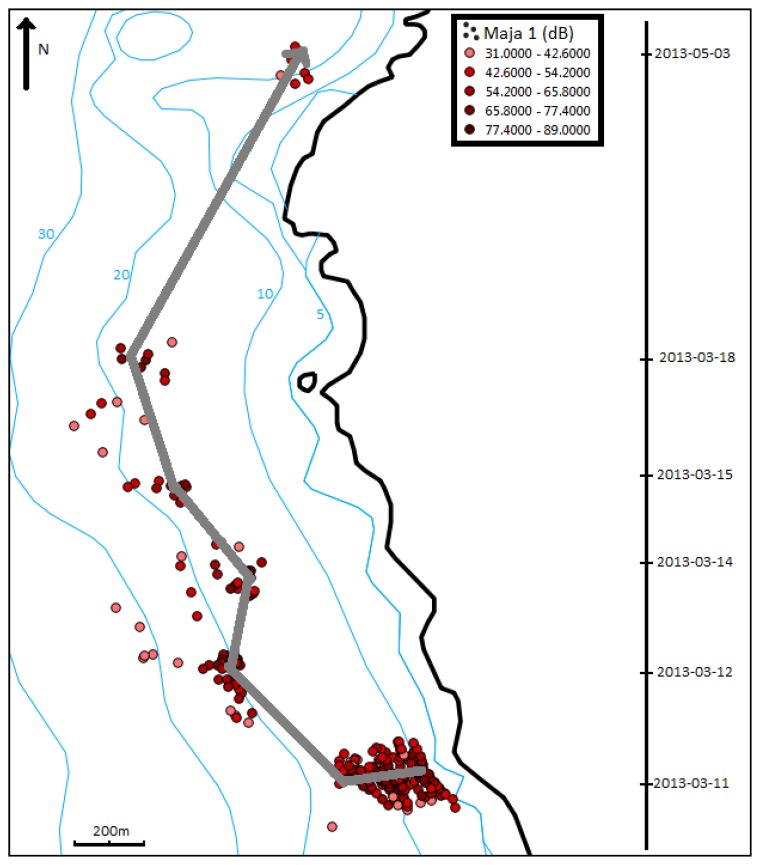
Trajectory of Maja 1 during the experimental campaign.

To perform this monitoring, two researchers went every day to the study site with a small boat. Due to the size and characteristics of the boat, and for safety reasons, it was impossible to track the spider crabs in severe weather and sea conditions. The results are illustrated by Figure 6. In Figure 6, we distinguish the different positions of the Maja 1 individual equipped with a V13TP. The color intensity of each point determines the signal strength. In Figure 6, we have represented the trajectory of Maja 1 according to the dates of recorded data during the two months.

Figure 6 shows an interesting result: the trajectory of Maja 1. Indeed we can observe a displacement along isobaths (blue lines). It is very important because that the choice of V13 transmitters and active tracking allowed us to monitor a part of the crab behavior. This result confirms that the technology can be used to study a species.

### 3.2. Limits of Our Work

The first results are interesting, however we have been limited by several aspects:

- The method is too costly in time and the amount of data collected is limited;
- We have equipped two individuals: Maja 1 and Maja 2. We had some problems to detect the signals of Maja 2 and the results are not really usable. According to the results obtained for Maja 2, this crab has moved of approximately 300 m in three days after release. Then we no longer noticed movement and we lost the signal six days later. We don't know if the spider crab 2 died, if she lost the transmitter and it was swept away by the current or if the transmitter burned out.

These limitations of our work have led us to build another system to respond to these problems.

### 3.3. Future Work

We have exposed the limits on this active tracking method and we a working on a new system according to several parameters:

- Passive tracking: deployment of drifting buoys according to a grid on the experimental area.
- Strategy of deployment of buoys: an optimization via simulation tool developed at the University of Corsica allows us to compute the ideal positions of the buoys according to several weighted criteria. These criteria can be bathymetry, administrative permissions and currentology of the studied area.
- Sensors: temperature, pressure, stream, brightness are important data in behavioral studies of a species and it seems important to complete our system with such environmental sensors.

## 4. Conclusions

The study of *Maja squinado* appears is strategic task in Mediterranean Sea for the development of the professional fishing activity and the relation with its environment. The **S**ustainable **TE**chnologies for **L**ittora**L A**quaculture and **MA**rine **RE**search (STELLA MARE) platform has the objective of removing some scientific barriers and allow a harmonious development of the fishing industry. This paper has focused on the study of the spider crab, *Maja squinado*, a symbol of deregulation of the fishing activity. The data on this species are not enough and we have decided to define a database for building a behavioral model. According to our active tracking on individuals equipped with V13 VEMCO sensors, we have collected some data on this behavior. We conducted six days of tracking over a period of two months and recorded 266 positions of Maja 1. We observed a displacement along a depth line during one month and a rise to 10 m in May (which corresponds with the reproduction period). We also observed that the *Maja squinado* remained in his home at night and moved during the day. Maja 1 traveled approximately 2.8 km during the study period. These first results are interesting, but they are not sufficient and we have decided to develop a complementary method: passive tracking on an experimental area, deployment of research tools for better monitoring, and multiplication of sensors to obtain more environmental data.

## Figures and Tables

**Table 1.** Characteristics of the tagged spider crab.

	**Sex**	**Size (cm)**	**Weight (kg)**	**TBWRA (%)**	**Tag**	**Monitoring Time (day)**
Maja 1	Female	178	1,474	0,81	V13TP	6
Maja 2	Female	156	1,102	1,08	V13TP	5

TBWRA: transmitter to body weight ratio in air.
